# Adult muscle-derived stem cells engraft and differentiate into insulin-expressing cells in pancreatic islets of diabetic mice

**DOI:** 10.1186/s13287-017-0539-9

**Published:** 2017-04-18

**Authors:** Violeta Mitutsova, Wendy Wai Yeng Yeo, Romain Davaze, Celine Franckhauser, El-Habib Hani, Syahril Abdullah, Patrice Mollard, Marie Schaeffer, Anne Fernandez, Ned J. C. Lamb

**Affiliations:** 1 Mammalian Cell Biology group, IGH CNRS, UM, UMR 9002, 141 rue de la Cardonille, 34396 Montpellier cedex 05, France; 2 Genetics & Regenerative Medicine Research Centre, Faculty of Medicine and Health Sciences, Universiti Putra Malaysia, 43400 UPM Serdang, Selangor Malaysia; 3 Networks and Rhythms in Endocrine Glands, IGF, CNRS UMR-5203, Montpellier, France

**Keywords:** Muscle stem cells, Beta-cell differentiation, Insulin secretion, Pancreatic islets

## Abstract

**Background:**

Pancreatic beta cells are unique effectors in the control of glucose homeostasis and their deficiency results in impaired insulin production leading to severe diabetic diseases. Here, we investigated the potential of a population of nonadherent muscle-derived stem cells (MDSC) from adult mouse muscle to differentiate in vitro into beta cells when transplanted as undifferentiated stem cells in vivo to compensate for beta-cell deficiency.

**Results:**

In vitro, cultured MDSC spontaneously differentiated into insulin-expressing islet-like cell clusters as revealed using MDSC from transgenic mice expressing GFP or mCherry under the control of an insulin promoter. Differentiated clusters of beta-like cells co-expressed insulin with the transcription factors Pdx1, Nkx2.2, Nkx6.1, and MafA, and secreted significant levels of insulin in response to glucose challenges. In vivo, undifferentiated MDSC injected into streptozotocin (STZ)-treated mice engrafted within 48 h specifically to damaged pancreatic islets and were shown to differentiate and express insulin 10–12 days after injection. In addition, injection of MDSC into hyperglycemic diabetic mice reduced their blood glucose levels for 2–4 weeks.

**Conclusion:**

These data show that MDSC are capable of differentiating into mature pancreatic beta islet-like cells, not only upon culture in vitro, but also in vivo after systemic injection in STZ-induced diabetic mouse models. Being nonteratogenic, MDSC can be used directly by systemic injection, and this potential reveals a promising alternative avenue in stem cell-based treatment of beta-cell deficiencies.

**Electronic supplementary material:**

The online version of this article (doi:10.1186/s13287-017-0539-9) contains supplementary material, which is available to authorized users.

## Background

Diabetes mellitus (DM) is identified by excessive blood sugar levels (hyperglycemia) that arises from the incapacity of pancreatic beta cells to supply the hormone insulin (reviewed in [1] and references therein). Type 1 diabetes mellitus (T1DM) results from autoimmune destruction of pancreatic endocrine beta cells (reviewed in [2–4]), whereas type 2 diabetes mellitus (T2DM) results from progressive exhaustion of beta cells due to acquired insulin resistance [5]. Insulin is secreted by beta cells present in histologically distinct and highly specialized structures in the pancreas—the islets of Langerhans. Pancreatic islets comprise four other cells types: glucagon-producing alpha cells, somatostatin-producing delta cells, pancreatic polypeptide-producing PP cells, and epsilon cells producing ghrelin, with alpha and beta cells representing the majority of islet cells.

The recent identification that stem cells can be derived to undergo lineage-specific differentiation has opened the possibility of stem cell-derived beta-cell replacement therapy in the treatment of T1DM (reviewed in [6]). Stem cells have been derived in vitro into functional beta cells for transplantation as nanodevices to restore insulin production after implantation either subcutaneously or behind the renal capsule (reviewed in [1, 7]). Beta cells have been obtained from embryonic stems cells (ESC) of both mouse [8] and human origin [9] (reviewed in [10]), or from induced pluripotent stem cells (iPSC) produced through direct transcriptional reprogramming using stemness transcription factor cocktails (reviewed in [10–13]), or through reprogramming via a lineage conversion strategy from fibroblasts [14]. Although still controversial [15], a number of studies have described techniques for inducing the beta cell-specific reprogramming of fibroblasts of multiple origins into glucose-responsive beta-like cells [16–18]. However, long-term approaches involving differentiation of iPSC and ESC are compromised by the latent potential of residual tumor-promoting stem cells to develop into teratomas or cause tumorigenesis [19]. While these techniques are tedious and time consuming [9, 13], they open the possibility that patient-derived iPSC may ultimately offer a viable source of functional beta cells to overcome the present difficulties in obtaining appropriate pancreas or islet beta-cell donors for transplantation [10]. Indeed in a recent study, differentiated beta cells derived in vitro from human iPSC were shown to secrete insulin in a glucose-responsive manner in vivo when transplanted and encapsulated in immunodeficient NOD-*Rag1* ^*null*^ *IL2rg* ^*null*^ *Ins2* ^*Akita*^ (NRG-Akita) mice and overcame progressively worsening hyperglycemia in these mice over several months [9]. However, attempts to restore normal glycemia after transplantation of differentiated beta cells into immunodeficient animal models of diabetes have only shown a short-term amelioration at best, likely due to the rapid destruction of the transplanted beta cells [11, 15].

As an alternative possibility, nontumorigenic adult stem cells may be directly transplanted into animal models of T1DM to investigate their ability to differentiate in vivo into functional beta cells. Such an approach was recently investigated using bone marrow-derived mesenchymal stem cells [20] and umbilical cord-derived mesenchymal stem cells [21].

The life-long regenerative and remodeling capacities of skeletal muscle make it a potential niche for multipotent adult stems cells (reviewed in [22, 23]). Human skeletal muscle growth and regeneration can be triggered by muscle damage or increased activity and exercise, and involves activation of quiescent stem cells to proliferate and differentiate into de novo muscle fibers, connective tissue, vascularization, and peripheral neural cells [22, 24]. We have previously isolated, via serial pre-plating, a population of nonadherent muscle-derived stem cells (MDSC) that can differentiate into smooth, skeletal, and cardiac muscle lineages, as well as neuronal lineages [25]. Although this multipotent differentiation implies an apparent heterogeneity of MDSC, like that of pluripotent ESC or iPSC, this heterogeneity is the signature of their multipotency as shown from similar adult muscle stem cells grown clonally [26] and revealing the expression of markers for the same multiple lineages as we described [25]. Here, we examined the potential of multipotent adult stem cells isolated from skeletal muscle (MDSC) to differentiate towards another lineage—insulin-producing beta cells. This study reveals that MDSC not only have the capacity to spontaneously differentiate into insulin-expressing and insulin-secreting clusters of beta-like cells in vitro*,* but also can be used directly in vivo without predifferentiation by direct intraperitoneal (IP) injection into mouse models of T1DM where they are recruited to pancreatic islets within 48 h and differentiate into insulin-expressing beta-like cells within 10 days of injection. Finally, we show that, in mice with streptozotocin (STZ)-induced diabetes, hyperglycemic levels are reduced after injection of undifferentiated MDSC (an effect not seen in mice injected with saline alone). Considering their rapid purification from skeletal muscle and the absence of any predifferentiation step, MDSC offer a unique and promising approach for autologous beta-cell replacement therapies.

## Results

### Cells extracted from skeletal muscle contain a nonadherent, nestin-enriched multipotent stem cell population—MDSC

**Fig. 1:**
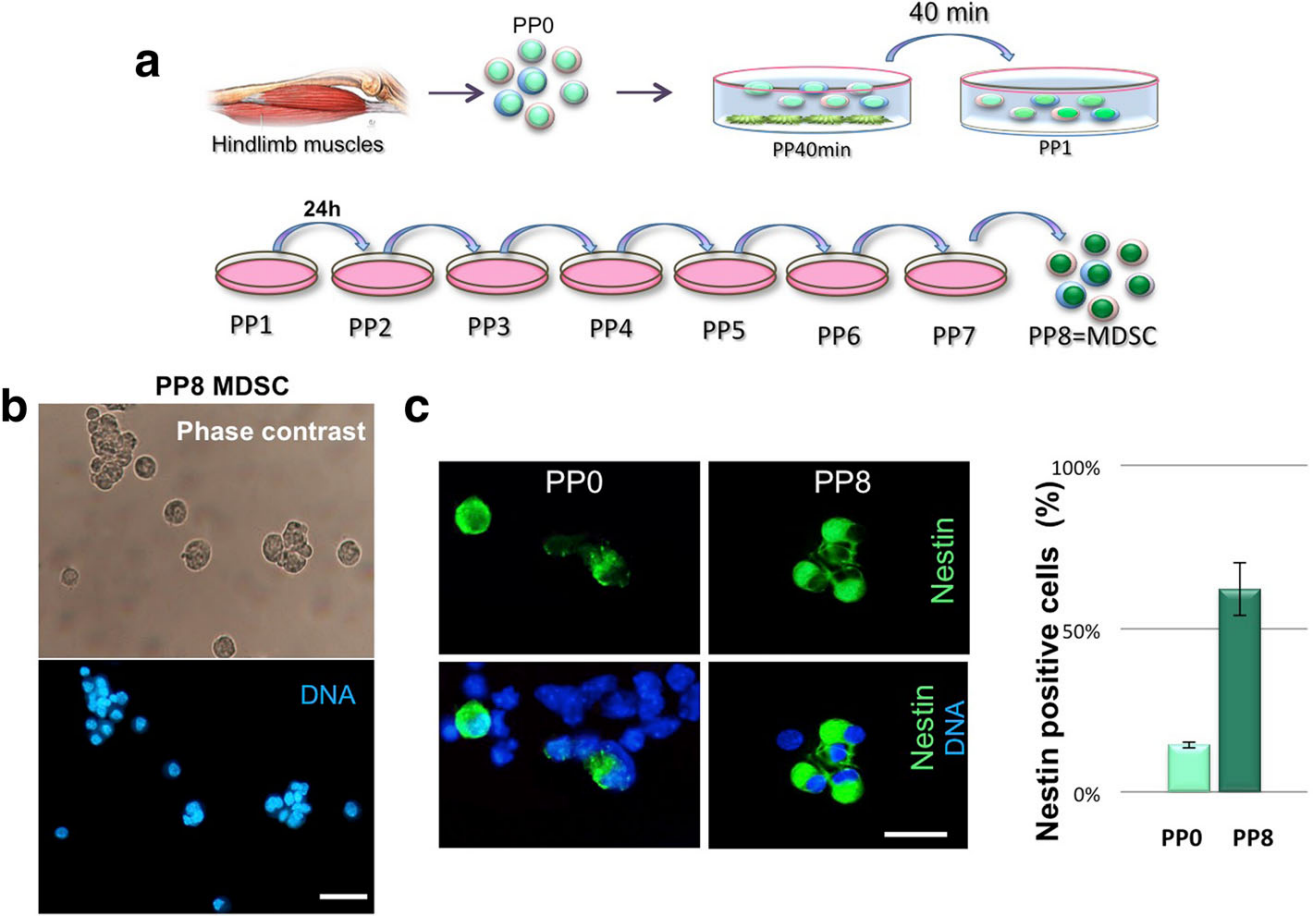
Schematic overview of the muscle-derived stem cell (*MDSC*) purification process and comparative analysis of nestin expression in PP1 vs PP8 cells. After a 40-min pre-plate to allow fibroblasts to adhere to the plate, nonadherent cells are daily re-plated in new medium and culture dishes over 7 days to produce a nonadherent and proliferative stem cell population—the MDSC. **a** Schematic diagram of the pre-plating purification process. **b** Representative photomicrographs of undifferentiated PP8-MDSC by phase-contrast and Hoechst staining for DNA. **c** Immunostaining analysis for nestin on cytospins of PP0 total muscle cells (*left panels*) and PP8 nonadherent MDSC (*right panels*). The histogram summarizes nestin expression in 300–400 cells for each sample (PP0 and PP8) performed after immunofluorescence analysis for nestin on five different MDSC isolation experiments. *Scale bars* = 20 μm

We have previously described a multipotent stem cell population derived from adult skeletal muscle based on the sequential pre-plating of nonadherent cells [25]. Figure 1a shows a schematic overview of the pre-plating purification process performed with “reversing” the spun culture media into the parental dish such that the nonadherent, floating MDSC fraction is always maintained in fresh, growth factor-enriched media. The typical phenotypes of MDSC maintained in a proliferative state after eight rounds of pre-plating are shown with phase contrast and DNA staining in Fig. 1b. At this stage, PP8-MDSC can either be amplified (by continued passage in high growth factor media) or allowed to spontaneously differentiate after seeding on laminin, on fibroblasts from early pre-plates, or on confluent, contact-inhibited fibroblast line (CCL-146) as a supply of extracellular matrix [25]. In the latter cases, MDSC spontaneously differentiate into several phenotypically distinct lineages including muscle cells, beating cardiomyocyte-like cells, and neuronal-like cells [25]. Figure 1c shows the immunofluorescence analysis for the neural stem cell marker nestin on PP0 muscle cells and PP8 MDSC and a comparison of the percentage of cells staining positively for nestin in total cells initially extracted from muscle (PP0) and in PP8 MDSC. This analysis reveals a fourfold increase in nestin-expressing cells by PP8 (from 16% in PP0 to 68% in PP8, average calculated from five different MDSC isolations with 300 to 400 cells counted for each sample). Nestin is a stem/progenitor marker shown to be expressed in progenitors of beta cells and present in pancreatic islets [27, 28]. This fourfold enrichment in nestin-expressing cells after the eighth pre-plating shows that the MDSC isolation procedure resulted in a significant enrichment in progenitor/stem cells.

### MDSC differentiate in vitro into beta islet-like cell clusters expressing insulin

**Fig. 2:**
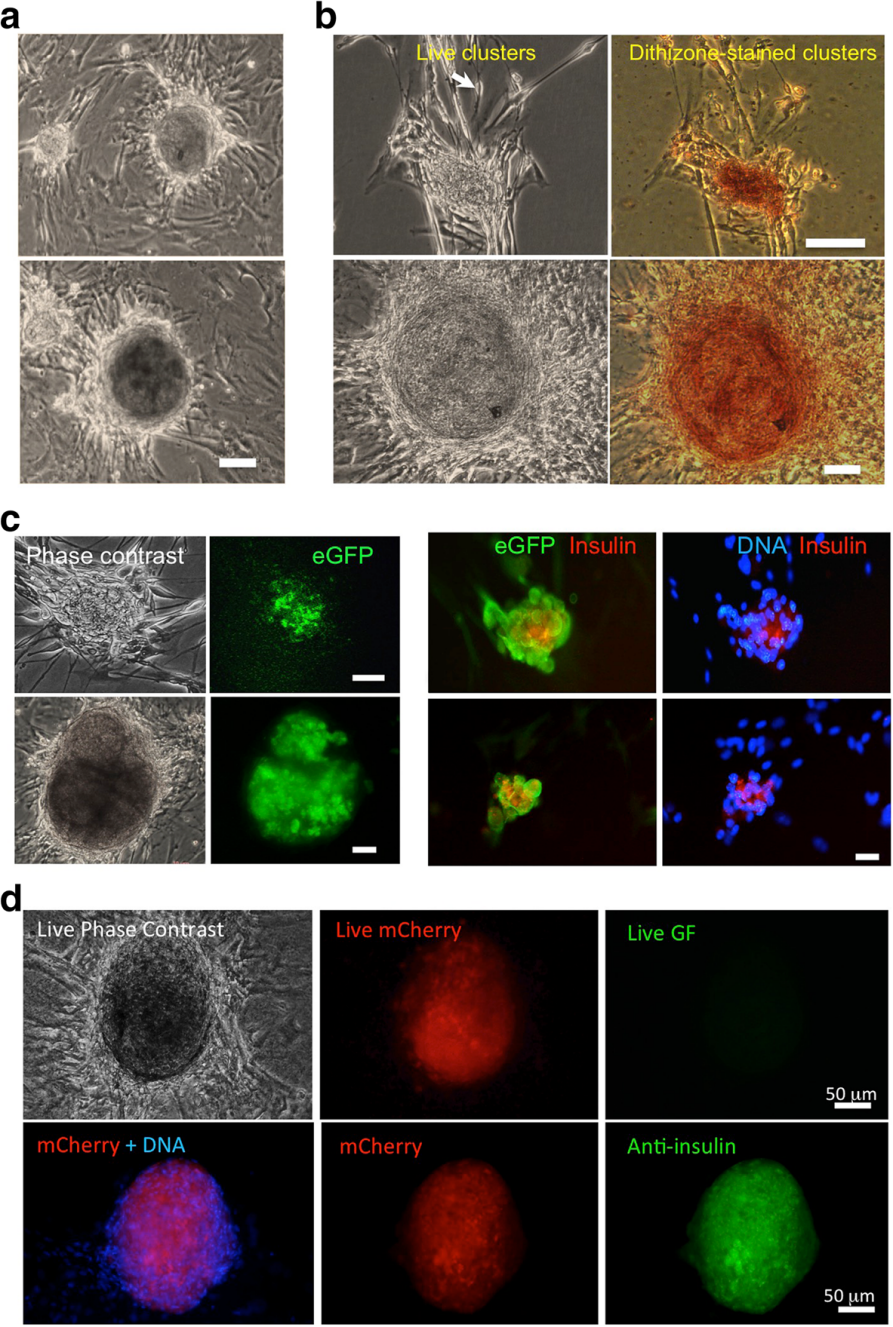
Long-term cultured MDSC spontaneously form cell clusters that stain positively for dithizone and express insulin. **a** Representative examples of small and large islet-like clusters spontaneously differentiated from 3-week cultured MDSC showing phase-contrast images of the living clusters. *Scale bar* = 50 μm. **b** Cell clusters staining positively for zinc using the reactive thiol reagent dithizone (DTZ), commonly used to identify insulin-expressing pancreatic beta cells. Shown are two different examples of DTZ-positive clusters from different preparations of MDSC. The *arrow* shows neuronal-like adherent cells associated with clusters but not stained with DTZ. *Scale bars* = 20 μm. **c** eGFP expression in live cell clusters derived from MIP-eGFP mice (*left panels*) and clusters immunostained for insulin (*red* in *right panels*). *Scale bars* = 20 μm. **d** Beta islet-like cluster differentiated from RIP-mCherry MDSC after 4 weeks. The top three panels show phase-contrast and live fluorescence of the cluster before fixation. The three bottom panels show mCherry (*red*) fluorescence with Hoechst staining for DNA and immunofluorescence staining for insulin (*green*) of the same islet-like cluster shown live in the three top panels

Pancreatic beta cells share high levels of transcriptional and functional similarity with neurons [29, 30]. In addition to enriched expression of nestin in PP8 MDSC (Fig. 1c), their differentiation into neuron-like cells [25] prompted us to investigate if MDSC could differentiate into pancreatic cells and, in particular, insulin-producing beta cells. As shown in Fig. 2a, PP8 MDSC seeded on adherent fibroblast cells from earlier pre-plates spontaneously differentiated after 3 weeks into three-dimensional cell clusters forming among neural-like cells. The size of these clusters varied, but they were clearly distinct from the surrounding flat fibroblasts and neural-like cells. In order to rapidly screen for insulin-positive cell aggregates and analyze a large number of cell clusters, we first stained living culture plates with dithizone (DTZ), a thioreactive stain that chelates zinc ions present in insulin-expressing pancreatic beta cells and a commonly used stain to identify pancreatic islet cells [31]. When MDSC were seeded onto adherent myofibroblast cells and allowed to differentiate for 14–21 days, several DTZ-stained cell clusters could be identified indicating that insulin-expressing cells may be present in clusters differentiated from MDSC (Fig. 2b). To confirm these data, we differentiated islet-like clusters from MDSC derived from a NOD-MIP-eGFP transgenic mouse model. In this mouse model, eGFP is expressed in pancreatic beta cells under the control of an exogenous murine INS1 promoter (Additional file 1: Figure S1). As shown in Fig. 2c (left panels), a group of cells present in islet-like clusters expressed eGFP indicating that the insulin promoter 1 was activated in these cells, thus reinforcing that the DTZ staining corresponds to effective insulin production in islet-like clustered cells. As expression of eGFP may vary significantly between different clusters, we next confirmed that the expression of eGFP corresponded with that of insulin by fixing eGFP-expressing clusters and staining in situ for the expression of eGFP and insulin. As shown in Fig. 2c (right panels), cells within the eGFP-positive cluster also expressed insulin as detected by immunofluorescence staining for insulin (stained in red), and all the insulin-expressing cells are also positive for eGFP as detected by anti-eGFP antibodies. The anti-eGFP staining also shows that a proportion of cells within the cluster are positive for eGFP but not insulin. These cells most likely represent beta-like cells that have expressed insulin and eGFP under the control of the insulin promoter and subsequently secreted insulin since, as shown below (Fig. 4c), MDSC-derived islet-like cell clusters secreted insulin in vitro. Similar data were also obtained using another transgenic mouse model expressing mCherry under the rat INS2 promoter (Additional file 1: Figure S1). As shown in Fig. 2d, live imaging of clusters formed after 1 month of differentiation of MDSC extracted from this mouse model also revealed endogenous mCherry fluorescence (red) and no staining in the green fluorescence channel. After the same cluster was fixed, the mCherry-positive beta-like cells present in the cluster also co-stained for insulin (Fig. 2d, bottom panels). These data confirm, using two different transgenic mouse models, that cultured MDSC spontaneously differentiated into insulin-producing beta-like cells within 2–4 weeks in vitro. In addition, a similar ability of MDSC to differentiate into islet-like cell clusters was also shown within 3–4 weeks of culture using MDSC from Wistar rat muscle (Additional file 2: Figure S2).

**Fig. 3:**
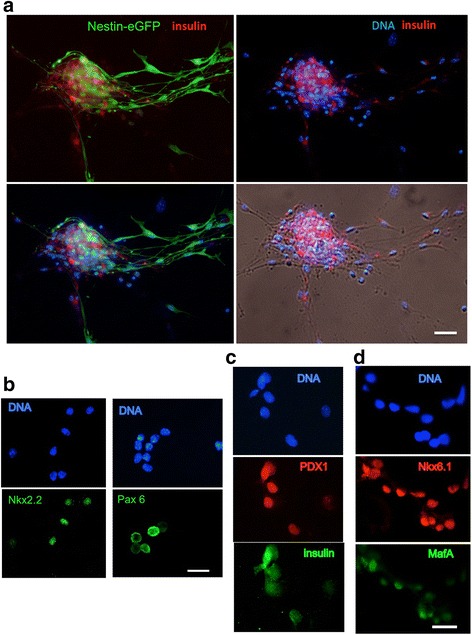
Immunofluorescence analysis of beta cell markers in islet-like cell clusters differentiated from MDSC. **a** Islet-like clusters were differentiated in vitro for 6 weeks from MDSC derived from nestin-eGFP transgenic mice. Photomicrographs show staining for eGFP and insulin (*red*) and DNA (*blue*). The bottom panels show a merged view of GFP, insulin, and DNA (*left panel*) and the phase-contrast image of the cluster with staining for insulin and DNA (*right panel*). *Scale bar* = 50 μm. **b**–**d** MDSC differentiated into islet-like clusters for 6 weeks were dissociated by short enzymatic treatment and collected by cytospin centrifugation, before fixation and staining for the expression of transcription factors implicated in beta-cell differentiation and insulin. Shown are fluorescence micrographs of dissociated cells stained for DNA and Nkx2.2 or DNA and Pax6 (**b**), co-stained for PDX1 and insulin (**c**), and for Nkx6.1 and MafA (**d**). *Scale bars* = (**b**) 20 μm, (**c,d**) 10 μm

We next examined if typical markers of progenitor and mature pancreatic beta cells ([32] and references therein) were expressed in beta-like cell clusters derived in vitro. In addition to its expression in neural progenitors, nestin-eGFP reporter expression was also shown to be expressed in the developing pancreas together with expression of both exocrine and endocrine markers [33]. As shown in Fig. 3a, using MDSC from a reporter nestin-GFP transgenic mouse [34], nestin-GFP was strongly expressed in differentiated beta islet-like clusters. As shown by co-staining for insulin (in red in Fig. 3a), nestin-expressing cells were surrounding but distinct from insulin-expressing cells in the islet-like cell cluster. Since nestin has been shown to be a stem/progenitor marker expressed in progenitors of beta cells and present in pancreatic islets [27, 28], the nestin-GFP-positive cells in the beta-like cell clusters differentiated from mouse nestin-GFP MDSC likely correspond to beta cell progenitors. The presence of beta cell-specific transcription factors was next examined, together with insulin expression, in enzyme-dissociated and cytospin-collected islet-like clusters from differentiated MDSC. Figure 3b shows that the homeobox Nkx2.2 and paired-homeobox Pax6 transcription factors were clearly expressed in dissociated cells, demonstrating that the cell clusters comprised cells differentiated into endocrine progenitors. Furthermore, co-immunostaining for the marker of functional mature beta cells MafA with Nkx6.1 and for the pancreatic duodenal homeobox gene 1 Pdx1 with insulin showed co-expression of MafA with Nkx6.1 (Fig. 3c) and of insulin with Pdx1 (Fig. 3d) in several cells dissociated from spontaneously differentiated islet-like cell clusters.

**Fig. 4:**
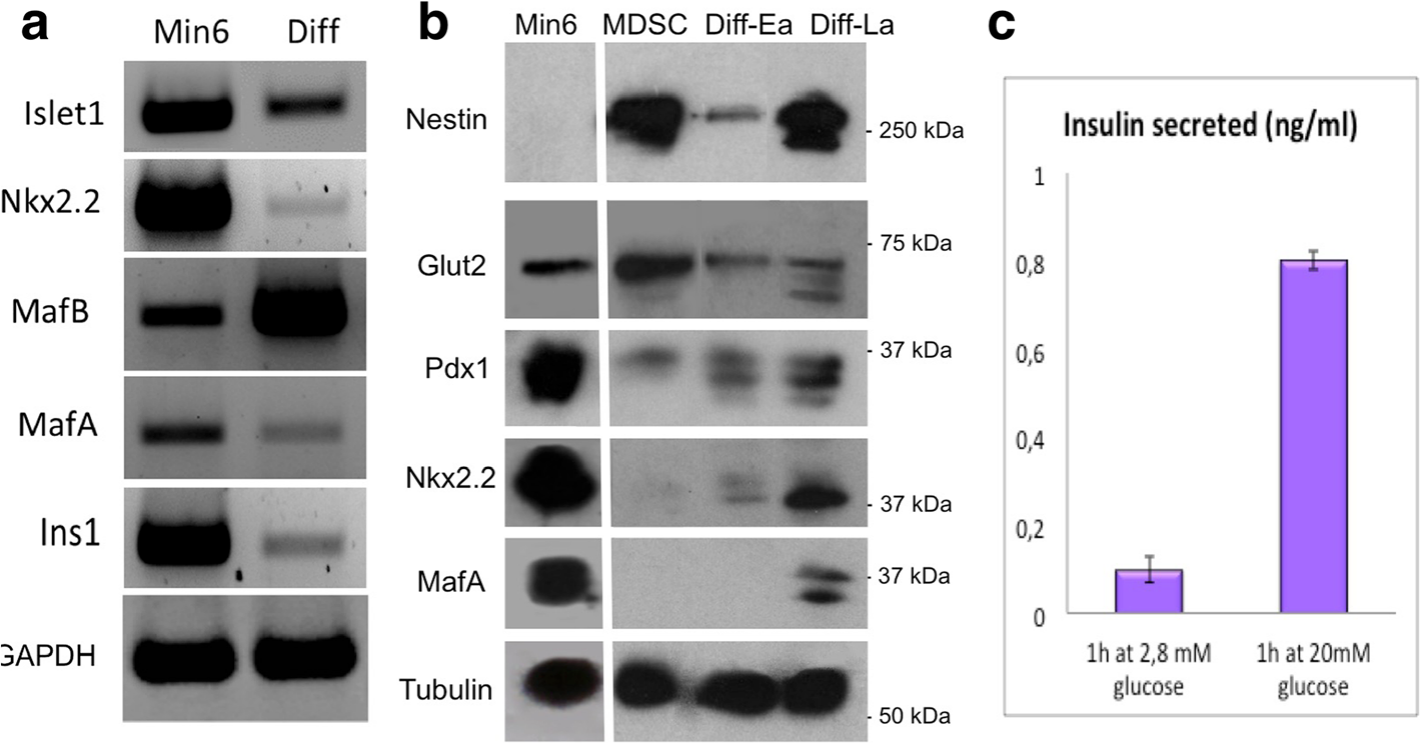
Islet-like clusters express specific markers of beta cells and secrete insulin in response to glucose challenge. **a** The mRNA expression profile of components implicated in the commitment, differentiation, and maturation of beta cells was examined in differentiated islet-like cell clusters by RT-PCR. Shown is the expression in 6-week differentiated (*Diff*) MDSC and in Min6 insulinoma cells as a control. **b** Protein expression profiles were also probed by Western blot analysis at three different time points,: undifferentiated PP8 MDSC (*MDSC*), early differentiated MDSC (*Diff-Ea*: PP8 + 2 weeks differentiation), and late differentiated MDSC (*Diff-La*: PP8 + 6 weeks differentiation). Shown are typical profiles blotted for Nestin, Glut2, Pdx1, Nkx2-2, and MafA. Alpha-Tubulin is shown last as a loading control. Molecular weight markers are indicated in kDa. **c** Glucose-responsive insulin secretion: for each assay, 4 to 5 islet-like cell clusters differentiated for 8–10 weeks were collected in 24-well dishes and starved in KREBS buffer for 2 h before incubating for 1 h in KREBS supplemented with 2.8 mM glucose and subsequently incubated for 1 h in 20 mM glucose. Secreted insulin was measured by FRET. Whereas little or no insulin was released in 2.8 mM glucose, when further challenged with 20 mM glucose, clusters secreted significant levels of insulin with regard to the small number of cells in the wells (1500 to 2000 cells/well, *n* = 3); *P < 0.01

Specific markers and transcription factors of beta cell development were also analyzed by RT-PCR for RNA expression of Islet1, Nkx2.2, and MafB (present in pancreatic development in endocrine progenitor stages and immature beta cells), and for the expression of insulin and MafA, both specific markers of mature beta cells. As shown in Fig. 4a, cells differentiated into islet-like clusters for 3–4 weeks in vitro revealed effective expression of islet1, Nkx2.2, insulin from the Ins1 gene, MafA, and MafB. The expression of insulin and the two transcription factors Nkx2.2 and MafA confirmed at the transcriptional level our observations by immunofluorescence (Fig. 3b and d). Finally, we examined by Western blot the expression of several components involved in differentiation and maturation of beta cells in order to obtain a quantitative assessment of their protein levels (Fig. 4b). For this analysis, expression levels were compared in undifferentiated PP8 MDSC, in MDSC differentiated for 2 weeks, (early differentiation (Diff-Ea)), and for 6 weeks (late differentiation (Diff-La)). Min6 insulinoma cell extracts were used as a positive control for markers of mature beta cells. The membrane was finally probed with anti-alpha tubulin as the sample loading control. This analysis first shows that nestin, which was expressed at high levels in undifferentiated MDSC (confirming the cytospin analysis in Fig. 1c), decreased during early differentiation before increasing again at the later differentiation stage in islet-like clusters. These blotted levels of nestin also confirmed the immunofluorescence data shown in Fig. 3a, where nestin (in green) was highly expressed in cells in the islet-like clusters. However, Fig. 3a also showed that insulin-expressing cells (in red) present in the same islet-like cluster were mostly distinct from nestin-GFP-expressing cells. In agreement with this, the Western blot for MIN6, that are fully differentiated beta cells, did not show any nestin expression. Significantly, markers of beta cell progenitors and mature beta cells all showed increased expression with the differentiation time in culture from Diff-Ea to Diff-La. In addition to the Glut2 glucose transporter protein, the major beta cell glucose transporter [35], the protein levels of Pdx1 and Nkx2.2 are also shown in Fig. 4b. Interestingly, both transcription factors were already detectable in MDSC and showed increasing levels after 2 and 6 weeks of differentiation. Late differentiated MDSC-derived islet-like clusters (Diff-La) also expressed the mature beta cell-specific transcription factor MafA, which was not detected in MDSC or at the early differentiation stages (Diff-EA).

While data from immunohistochemistry, RT-PCR, and Western blot all show that the MDSC differentiated clusters expressed markers of immature and mature beta cells, it remained to be shown if they also secreted insulin in response to a glucose challenge. Several reports have demonstrated that different stem cells can be differentiated into mature insulin-secreting beta-cells in vitro [15–17], but only relatively few have shown that these cells effectively secrete insulin in a glucose-responsive manner [9, 20, 36, 37] (reviewed and discussed in [1, 6, 7, 38]). We therefore set up a standard insulin secretion assay using clusters differentiated in vitro for 8–10 weeks. For each measure, 4 to 5 islet-like cell clusters were collected in 24-well dishes and starved in KREBS buffer for 2 h before incubating for 1 h in KREBS supplemented with 2.8 mM glucose and subsequently incubated for 1 h in 20 mM glucose. Secreted insulin was measured by a FRET-based detection kit (CISBIO). Figure 4c shows that, when clusters were incubated for 1 h in 2.8 mM glucose, little or no insulin secretion was detected. In contrast, further stimulation for 1 h in 20 mM glucose resulted in a robust and consistent increase in insulin secretion (0.8–1 ng/ml of secreted insulin). Indeed, clusters secreted significant levels of insulin with regard to the small number of cells in the beta-like clusters (1500 to 2000 cells/well). Assays were performed in triplicate and similar results were obtained using at least three different preparations of MDSC-derived islet-like clusters, all showing effective insulin secretion in response to a 20 mM glucose challenge.

Taken together, these data show that both mouse and rat MDSC have the capacity to spontaneously differentiate in vitro into three-dimensional cell clusters that bear many of the hallmarks of mature pancreatic beta cells, and that beta islet-like cell clusters are competent to secrete insulin in a glucose-responsive manner.

### In vivo homing to pancreatic islets and differentiation of MDSC into insulin-expressing beta cells in STZ-induced diabetic mice

**Fig. 5:**
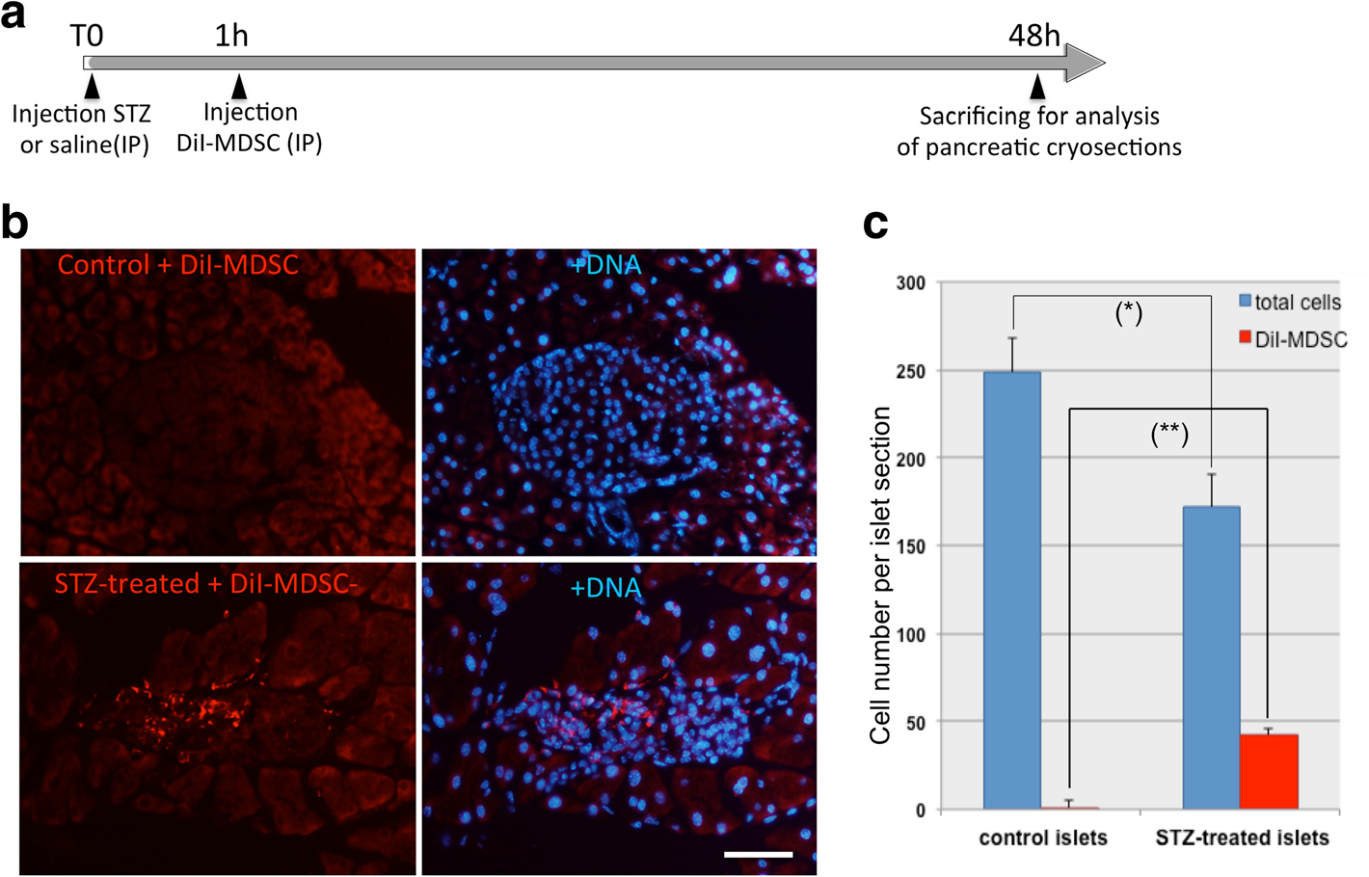
Muscle-derived stem cell (*MDSC*) engraftment in pancreatic islets from streptozotocin (*STZ*)-treated mice 48 h after intraperitoneal injection (*IP*). **a** PP8 MDSC labeled with the cell tracker membrane dye DiI were administered by IP injection into mice 1 h after IP injection of either saline solution (control) or streptozotocin (STZ treated), and the pancreas was removed for cryosection analysis 48 h later. **b** Shown are representative fluorescence micrographs of sections from untreated control (*upper panels*) and STZ-treated mice (*bottom panels*). *Left panels*, DiI fluorescence (*red*); *right panels*, Hoechst staining for DNA (*blue*). *Scale bar* = 100 μm. **c** Islet engraftment of DiI-labeled cells in STZ-treated and untreated mice in comparison to total cell nuclei counted per islet in the pancreatic cryosections analyzed. Represented in the histogram are the average number of cells counted per islet section and DiI MDSC engrafted in control and STZ-treated islets. *P < 0.01, **P < 0.001

Having determined the capacity of MDSC to differentiate into beta-like insulin-secreting cells in vitro, we next investigated if undifferentiated MDSC could also differentiate towards a beta cell lineage in vivo after injection in living mice with chemically induced T1DM. The commonly used model involves treating 4- to 6-month-old male mice with the glucose analogue streptozotocin (STZ), either at a single high dose or following a 5-day treatment with a lower dose [39]. Intraperitoneal (IP) injection of STZ is followed by its rapid and toxic uptake into pancreatic beta cells via Glut2. Our initial experiments examined whether MDSC labeled with the membrane-bound cell-tracker dye CM-DiI would localize in the pancreas differently between untreated and STZ-treated mice. Figure 5a shows the timeline of the experiment: 1 h after IP injection of STZ or saline, mice were injected with CM-DiI-labeled MDSC IP and, 48 h after, mice were sacrificed and the pancreas removed and processed for cryosection analysis. Figure 5b (top panels) shows a typical cryosection from the pancreas of saline only and MDSC-injected mouse with a clearly delineated islet (staining in blue for DNA) in which no DiI-labeled cells are detected. In contrast (Fig. 5b, lower panels), the poorly delineated islet-like structure visible in the pancreatic section of a STZ-treated and MDSC-injected mouse clearly contains DiI-labeled MDSC, confirming that within 48 h DiI-labeled MDSC have homed effectively to the STZ-damaged islets. This islet-specific engraftment of DiI-labeled cells in STZ-treated mice was confirmed in all the islets analyzed in the pancreatic cryosections. The average number of cells counted per islet section is shown in Fig. 5c: 250 cells/control islet versus 170 cells/STZ-treated islet with no DiI donor cells in control versus >40 DiI donor cells/islet in STZ-treated islets. These data indicate that, upon systemic IP injection, MDSC distribute nonrandomly with preferential engraftment to STZ-damaged beta pancreatic islets. Such specific recruitment of MDSC to damaged beta islets was also observed with other types of organ and tissue damage, and likely reflects the capacity of MDSC to migrate and home to tissue niches harboring inflammatory processes (Mitutsova et al., manuscript in preparation).

**Fig. 6:**
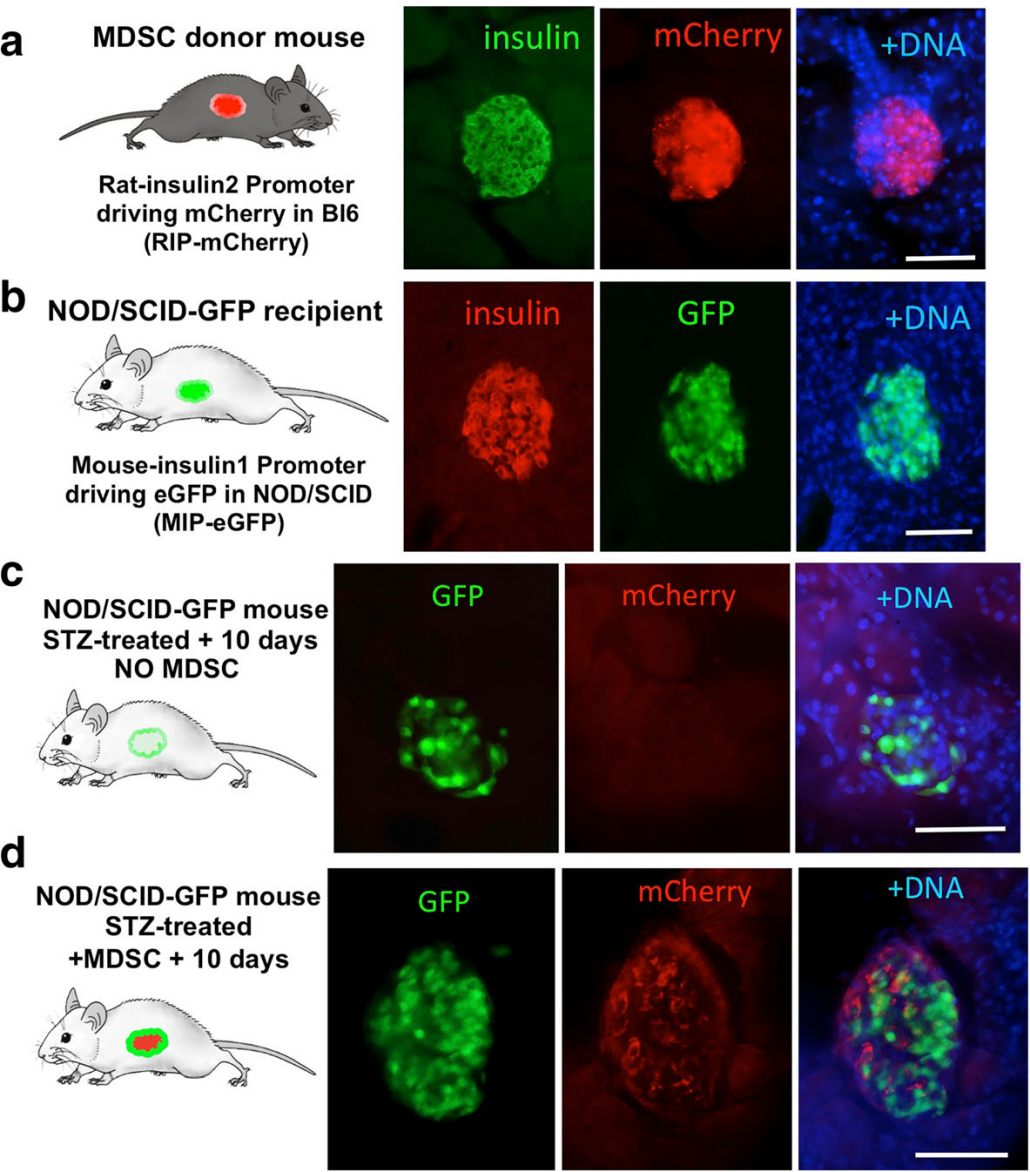
Muscle-derived stem cells (*MDSC*) differentiate in vivo into insulin-expressing cells 10 days after intraperitoneal injection in streptozotocin (*STZ*)-treated mice. To examine if undifferentiated MDSC could differentiate in vivo into functional beta cells, MDSC were isolated from a donor mouse expressing mCherry under the control of the rat INS2 promoter that expressed mCherry specifically in pancreatic beta cells as shown by co-immunostaining for insulin (*green*) (**a**). Recipient NOD-SCID-MIP-eGFP transgenic mice expressed eGFP under the control of the mouse INS1 promoter specifically in pancreatic beta cells as shown by co-staining for insulin (*red*) (**b**). Recipient MIP-eGFP mice were injected with the glucose analog STZ to destroy endogenous beta cells and 60 min later with either saline vehicle (STZ only) or MDSC (STZ + MDSC), and pancreatic vibratome sections were analyzed 10 days later. Panels in (**c**) show staining for eGFP (*green*) and mCherry (*red*, no signal) in a pancreatic islet of MIP-eGFP mouse 10 days after STZ injection alone. Panels in (**d**) show staining for eGFP (*green*) and mCherry (*red*) in an islet of MIP-eGFP mouse 10 days after injection of STZ and undifferentiated muscle stem cells from a RIP-mCherry donor mouse. *Scale bars* = 100 μm

In order to examine whether MDSC could differentiate into pancreatic beta cells in vivo after engraftment within 48 h into STZ-damaged islets (Fig. 5), we exploited the availability of the two transgenic mice models described in Additional file 1: Figure S1 and Fig. 6a and b. Mice expressing mCherry under the control of the rat INS2 promoter (RIP-mCherry) were used as donors for MDSC (Fig. 6a), and NOD-scid-MIP-eGFP mice in which eGFP expression is under the control of the mouse insulin promoter were used as recipient mice (Fig. 6b). Insulin expression (in green) in the pancreatic islets of the RIP-mCherry mouse correlated with the expression of the mCherry reporter (shown in red; Fig. 6a), whereas insulin expression (in red) in the islets of the MIP-eGFP mouse correlated with the expression of the eGFP reporter (Fig. 6b). In both cases it should be noted that the fluorescent reporter co-expressed but, as expected, did not co-localize with insulin. We isolated MDSC from RIP-mCherry donor mouse muscle, which were then injected IP as undifferentiated stem cells into NOD-scid-MIP-eGFP mice that had been treated with STZ to specifically destroy beta cells. Due to the immunosuppressed *scid* background in the recipient mice (NOD-scid-MIP-eGFP), we anticipated that the different genetic background of the donor mice (Black6) would not initiate an effective immune response against MDSC donor cells. Shown in Fig. 6c are sections through the pancreas of recipient mice 10 days after STZ treatment and injection of either vehicle only (0.9% saline; Fig. 6c) or undifferentiated MDSC derived from RIP-mCherry mice (Fig. 6d). In mice injected with vehicle only, there was a marked reduction in the levels of eGFP-expressing beta cells in pancreatic islets (compared with GFP in Fig. 6b) and no signal for mCherry. In contrast, in recipient STZ-treated mice injected with undifferentiated MDSC from mCherry donor mice (Fig. 6d), the donor MDSC have differentiated in the pancreatic islets as revealed by expression of mCherry in recipient NOD-scid-MIP-eGFP mouse islets, indicating that donor MDSC have differentiated in vivo into insulin-expressing beta-like cells.

These data show that donor stem cells were retrieved in the pancreatic islets of STZ-treated mice within 2 days and differentiated into insulin-expressing beta cells after 10–12 days. Therefore, in addition to their in vitro capacity to differentiate into islet-like cells, undifferentiated mouse MDSC can also differentiate in vivo into beta-like insulin-expressing cells within pancreatic islets of mice with STZ-induced beta-cell destruction.

**Fig. 7:**
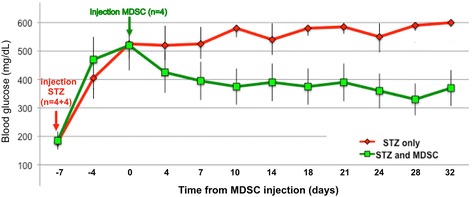
Blood glucose levels in streptozotocin (*STZ*)-induced diabetic mice injected intraperitoneally with or without muscle-derived stem cells (*MDSC*). Six-month-old male C57Bl6 were injected intraperitoneally with a solution of STZ at 150 mg/kg and blood glucose levels were measured at the tail tip twice per week at 2 pm (at resting day time). After 7 days, when blood glucose levels had risen above 450 mg/dl, half the mice (*n* = 4) were injected intraperitoneally with 300,000 MDSC in 200 μl saline solution prepared from consanguineous female Bl6 mice and the other half were injected with 0.9% NaCl solution only (*n* = 4). Shown are the average blood glucose levels measured and plotted for each time point revealing a decrease of the glycemia in MDSC-injected mice by 7–10 days after intraperitoneal injection of the stem cells

We finally examined if this observed homing, engraftment, and differentiation of MDSC in the pancreatic islets of STZ-treated mice could be accompanied by improved levels of blood glycemia. Figure 7 shows the blood sugar levels of 6-month-old male C57Bl6 mice after IP injection of 150 mg/kg STZ. Blood glucose levels were measured at the tail tip twice per week. After 6–7 days, when blood glucose levels were raised to hyperglycemia, half of the mice (*n* = 4) were injected IP with MDSC and the other half were injected with 0.9% NaCl vehicle solution only (*n* = 4). Shown are the average blood glucose levels measured and plotted for each time point, revealing a significant decrease of the glycemia in MDSC-injected mice from 10–28 days after IP injection of the stem cells. While further work involving a larger number of recipient STZ-treated mice will be required to fully establish the insulin secretory phenotype and responses after engraftment of MDSC cells in beta cell-deficient animal models, these data strongly suggest that MDSC are a suitable source of stems cells for pancreatic beta cell replacement therapies.

## Discussion

Here we have shown that a population of multipotent progenitors isolated from skeletal MDSC are competent to differentiate in vitro into insulin-expressing and secreting islet-like cell clusters. These cell clusters express a number of typical markers of endocrine differentiation, pancreatic progenitor, and mature pancreatic beta-cell differentiation. The islet-like cell clusters differentiated in vitro expressed insulin as confirmed with fluorescent reporters (eGFP or mCherry) in clusters formed from MDSC extracted from MIP-eGFP or RIP-mCherry transgenic mouse muscles and also secreted insulin in response to a glucose challenge. Strikingly, when assayed in vivo*,* after MDSC freshly purified as stem cells were injected IP into mice with chemically induced beta-cell destruction, they specifically localized to damaged islets within 48 h and differentiated into insulin-expressing beta cells within 10 days of injection. In addition, injection of undifferentiated MDSC into hyperglycemic mice effectively reduced their blood glucose level towards normal levels.

Whereas mesenchymal stem cells derived from bone marrow and defined as a stromal cell population have been extensively investigated [13, 20, 21], the muscle stem cell population we have examined here was isolated and purified on the basis of its lower adherence to plastic and even collagen-coated dishes [25]. Such low adherence properties may favor a higher migration capacity after systemic injection, and skeletal muscle displays a number of essential characteristics that make it a potentially ideal source of stem cells for direct use in vivo*.* With a life-long self-renewal capacity to repair damage or to undergo de novo growth and differentiation, skeletal muscle is remarkable by its paucity to develop cancer except in rare cases of rhabdomyosarcoma in childhood (reviewed in [40, 41]). This high plasticity of skeletal muscle, capable of rapidly undergoing atrophy or hypertrophy throughout life, involves the regeneration of different types of tissue and organs in addition to muscle fibers: connective tissue, vascularization, and, most importantly, innervation (since a denervated muscle cannot fully regenerate and instead undergoes atrophy [42]). The intermediate filament nestin, known to be a marker of meso-ectodermal stem and progenitor cells, is present in 12 to 15% of the cells initially isolated from muscle tissue and enriched fivefold (to 75–80%) during the MDSC purification process (Fig. 1c). Nestin is not only a marker of neuronal progenitor and stem cells [34] but is also expressed in progenitors cells present in pancreatic islets [8, 27, 28]. In addition, when pancreatic progenitors and insulin-expressing cells are derived from mouse ESC, nestin is expressed during the progenitor phase [43] and is shown to play essential roles in the process of stem cell differentiation into insulin-secreting cells [44]. When insulin-expressing clusters were differentiated from MDSC expressing eGFP under the control of the nestin promoter, we observed that nestin-GFP-positive cells accumulated in and around islet-like clusters together with, but distinct from, insulin-expressing cells (Fig. 3a). We have previously shown that MDSC are multipotent and can differentiate into at least five different meso-ectodermal-derived lineages including typical neuronal-like cells [25] and automatically beating pacemaker cells (Mesirca et al., manuscript in preparation). Here we show that, in addition to these two excitable cell types, MDSC can also spontaneously differentiate within 2–3 weeks in culture into cell clusters that express several markers of another excitable cell type, the pancreatic beta cell lineage.

While several studies using pluripotent stem cells have shown their potential for differentiating into insulin-producing beta cells, including via ectodermal commitment [8, 11, 31, 45, 46] (reviewed in [1, 13, 14, 35]), few have shown a short-term ability of insulin-producing beta-like cells to decrease STZ-induced hyperglycemia in vivo when transplanted into encapsulated structures in immunodeficient mouse models of T1DM [9, 20] (reviewed in [13, 14]). Most studies have exploited relatively complex differentiation protocols lasting for 4–6 weeks and involving four or five distinct steps [13]. In vitro, cultured MDSC spontaneously differentiated into insulin-expressing and -secreting beta-like cell clusters a few weeks after stem cell isolation and culture. These data add beta islet cells as a therapeutically important differentiation lineage to the expanding repertoire of cell types that can be derived from the MDSC multipotent muscle stem cell population [25].

Our principal objective, however, was to investigate the ability of MDSC to *differentiate* in vivo because undifferentiated stem cells retain greater plasticity, survival, and migration capacity. Indeed, there is growing evidence that mesenchymal stem cells which can be isolated from several adult tissues including bone marrow and adipose tissue (reviewed in [1, 6]) and neural crest-derived multipotent stem cells present in all peripherally innervated tissues (reviewed in [47]) may represent important cell sources for regenerative therapy. We have investigated here the in vivo behavior of undifferentiated MDSC injected into a mouse model of beta-cell deficiency induced by streptozotocin treatment. The nonteratogen character of MDSC (for up to 6 months follow-up after subcutaneous or IP injection into immunodeficient mice; VM, AF, and NJCL, unpublished observation) does not require their predifferentiation before use in vivo, and MDSC were transduced directly via IP or intravenous injection. We have examined their biodistribution within 48 h afterwards and found that MDSC are recruited to site(s) of injury, be it mechanical or chemically induced (Mitutsova et al., manuscript in preparation). Indeed undifferentiated purified MDSC (PP8) loaded with an in vivo cell tracer and directly transduced by IP injection were retrieved 48 h later in the beta cell-depleted pancreatic islets of STZ-treated mice and subsequently differentiated into insulin-expressing beta cells. The rapid islet-specific engraftment of MDSC after their IP injection brings support to an original report by Kozlova and Jansson showing that the migration and differentiation of neural crest stem cells are stimulated by pancreatic islets both in vitro and in vivo [48]. We believe that this is the first example of specific homing of undifferentiated adult stem cells to damaged pancreatic islets and their subsequent differentiation into insulin-producing beta cells. In addition, MDSC were purified by a relatively fast and simple protocol using standard media and techniques, which are both positive aspects for a potential future therapeutic use. It remains to be determined if in vivo differentiated MDSC secrete sufficient insulin to durably restore fully normal glycemia levels in STZ-induced T1DM mouse models. Despite the high variability of the response to STZ amongst recipient mice [39], and known endogenous beta islet cell regeneration [7] and islet plasticity [49] in addition to the variable immunogenic response against MDSC after they differentiated into beta-like cells, our preliminary experiments in mice rendered hyperglycemic after a single high dose of STZ showed a partial reduction of the glycemia from 1 to 3 weeks after injection of MDSC (Fig. 7). Although encouraging, this result will require further confirmation to fully establish the insulin secretory phenotype and responses after engraftment of MDSC cells in beta cell-deficient animal models.

## Conclusion

Overall, we show here that skeletal muscle-derived stem cells (MDSC) can undergo effective differentiation into insulin-expressing and -secreting beta-like cells both after in vitro culture and in vivo after systemic injection into mice with STZ-induced pancreatic beta cell destruction. The in vivo study represents, to our knowledge, the first demonstration of specific engraftment and differentiation of stem cells in pancreatic islets of diabetic mice. These data open a feasible therapeutic route through the potential use of MDSC by direct autologous injection of patient-derived stem cells as a promising alternative to the transplantation of differentiated and encapsulated beta cells.

## Methods

Unless specified, all chemicals were obtained from Sigma-Aldrich, Saint-Quentin Fallavier, France. Cell tracker DiI (dioctadecyl-3-tetramethylindocarbocyanine perchlorate) was the chloro-methylbenzamido-DiI analog, CM-DiI, from Thermo-Fischer (Villebon-sur-Yvette, France) and used according to the manufacturer’s instructions.

### Animals

Mice lines C57Bl6-wild type, NOD-MIP-eGFP (NOD/ShiLtJ-Tg(Ins1-EGFP/GH1); JAX-005282), and NOD-scid (NOD.CB17-Prkdc^*scid*^/J: JAX-001303) were from Jackson Labs (Bar Harbor, ME, USA), Black6-RIP-mCherry [50] and Nestin-GFP were from Dr. G. Enikolopov (Cold Spring Harbor Laboratory, NY, USA) [34]. NOD-scid-MIP-eGFP mice were obtained by crossing NOD-scid and NOD-MIP-eGFP. Sprague Dawley rats were from Charles River laboratory, and all animals were maintained in the IGH/IGF transgenic mouse facility. All animal handling strictly conformed to university, local, and international animal husbandry, veterinary care, and ethics rules (see below).

### Cell culture

Gerbil fibroma fibroblasts (CCL-146) were maintained in DME supplemented with 10% v/v serum as described previously [51]. MIN6 cells were cultured in DMEM (Gibco-LifeSciences, ThermoFischer, Villebon-sur-Yvette, France) supplemented with 25 mM glucose, 16% fetal bovine serum (FBS; Pall-Biosepra, Cergy-Saint-Christophe, France), penicillin/streptomycin (Gibco), and 55 μM β-mercaptoethanol (BME).

### Antibodies

Unless stated otherwise, antibodies were obtained from the Developmental Studies Hybridoma Bank (DSHB, Iowa, USA); for IHC and Western blotting: anti-eGFP chicken polyclonal GFP-1020 (Aves Inc, Tigard, OR, USA; 1/500); mouse monoclonal against: insulin (I2018; Sigma; 1/1000), Nestin (Rat-401; 1/200), PDX1 (F109-D12; 1/500), Nkx6.1 (F55-A10; 1/200), Nkx2.2 (74.5A5; 1/200), Pax6 (PAX6; 1/500); rabbit polyclonal antibodies against: MafA (A300-611A; Bethyl-Laboratories, Montgomery, Texas, USA) and Glut2 (H-67; Santa-Cruz Biotechnologies, CliniSciences, Nanterre, France).

### Isolation and purification of MDSC

After surgical removal, hind leg muscles were subject to enzymatic dissociation (0.2% (w/v) collagenase A (Roche, Basel, Switzerland) and 1 mg/ml dispase (Gibco) for 45 min), the crude muscle cell-containing fraction was isolated by filtration and centrifugation essentially as described before [25]. Subsequently, after a short 40-min incubation to allow fibroblasts to adhere, the MDSC-containing supernatant was recovered and MDSC collected by centrifugation (576 × g, 10 min) at room temperature (RT). Pellets were resuspended in proliferation media (PM; DME/Hams F12 mixture, supplemented with 16% (v/v) fetal calf serum (FCS; Gibco), 1% Ultroser G (Pall-BioSepra), 2 mM l-glutamine, antibiotic/antimycotic mix (Gibco), and 100 μM BME (added freshly from a 100 mM stock in phosphate-buffered saline (PBS)) and plated at a density of 2.5 million cells/100-mm dish as described previously [25]. After 24 h, unattached cells were gently detached by pipetting and collected by centrifugation (576 × g, 10 min). Pelleted cells were resuspended in 10 ml fresh media and transferred to a new 100-mm dish. MDSC were purified by eight rounds of this plating technique performed every 24 h which ensures that the nonadherent MDSC fraction is always passed daily into fresh growth factor-rich media. Figure 1a shows a schematic overview of the pre-plating process and typical examples of the different cell phenotypes that arise after MDSC from PP8 are allowed to adhere (on adherent cells from previous pre-plates, inactivated CCL-146 fibroblasts, or laminin-coated dishes [25]) and differentiate for 1 to 3 weeks. Exactly the same protocol was followed for producing rat MDSC.

### RT-PCR

For RT-PCR analysis of stem cells, MDSC were obtained after seven serial pre-plates (PP8). For differentiated cell clusters, MDSC (PP8) were seeded on matrix-providing adherent cells of earlier pre-plates or inactivated fibroblasts (CCL-146) and islet-like clusters formed 15–20 days thereafter. Clusters were picked from adherent cell monolayers (8 to 12 clusters 6 weeks after plating) and total RNA was extracted using RNeasy (Quiagen, Germany). cDNA was generated using SuperScript III Reverse Transcriptase (Invitrogen, ThermoFischer, Villebon-sur-Yvette, France) according to the manufacturer’s protocol using 50 nM oligo(dT)_20_. PCR was performed for 35 cycles at an initial denaturing temperature of 94 °C for 3 min, denaturing temperature of 94 °C for 45 s, annealing temperature of 55 °C for 30 s, extension temperature of 72 °C for 90 s, and final extension at 72 °C for 10 min using GoTaq polymerase (Promega, Charbonnières-les-Bains, France).

### Western blot analysis

For protein analysis, proteins were isolated, separated by SDS-PAGE, and analyzed by Western blot on PVDF membranes as described previously [52]. Briefly, nonadherent cells (PP8) were washed once in ice-cold PBS and collected by centrifugation at 570 × g for 10 min before freezing as pellets until use. Adherent differentiated cells, including clusters, were washed once in ice-cold PBS and, after manual detachment, collected by centrifugation (570 × g for 10 min) before freezing as pellets until use. Frozen pellets were resuspended in 40 mM Tris-Cl pH 6.8, 1.0 mM EDTA, 1% (w/v) SDS, and 7.5% (v/v) glycerol, and cell DNA was sheared by 10 passages through a 26G needle, cleared by brief centrifugation, and the protein concentration of the supernatant determined by Lowry assay (BioRad, Mitry-Mory, France). Subsequently, samples were diluted with an equal volume of 200 mM Tris-Cl pH 6.8, 4.0 mM EDTA, 4% (w/v) SDS, 30% (v/v) glycerol, 400 mM DTT, and 0.03% (w/v) bromophenol blue. Ten to 15 μg total protein/lane were resolved on 12.5 or 10% SDS-PAGE gels and transferred to PVDF-immobilon (Millipore, Merck, Fontenay sous Bois, France). Following transfer, membranes were stained with amido-black to confirm loading homogeneity before blocking by incubation in 1% (w/v) PVP for 15 min followed by 30 min incubation in TBS containing 0.5% (w/v) BSA and 0.5% (w/v) skimmed milk. Membranes were incubated with primary antibodies overnight at 4 °C in the same buffer.

### Insulin secretion

Insulin secretion was assayed by FRET-based assay using an insulin homogeneous time resolved fluorescence (HTRF) kit (Cisbio International, Codolet, France) according to the manufacturer’s instructions. Twenty-four hours before the assay, islet-like clusters were transferred to low-glucose DME (5.6 mM, 1 g/l). The next day, islet-like cell clusters (4–5 clusters) were transferred to 24-well plates and washed 3× with 1 ml Hanks balanced salt solution and then starved in 250 μl sterile filtered Krebs-Ringer solution (KR; 119 mM NaCl, 4 mM KCl, 1.2 mM KH_2_PO_4_, 1.2 mM MgSO_4_, 2.5 mM CaCl_2_, 20 mM HEPES, 5 mM NaHCO_3_ pH 7.5, and 0.1% (w/v) BSA Fraction 5 ultra-pure (Sigma-Aldrich)) for 2 h. After starvation, the KR solution was collected (T0) and replaced by 250 μl KR supplemented with 2.8 mM glucose, and clusters were incubated for 1 h. After collection of the ringer solution (T2.8), cells were washed 3× with KR before transfer to KR supplemented with 20 mM glucose and further incubated for 1 h (T20). Culture supernatants were stored frozen at –20 °C until analysis. Insulin released during incubation was quantified in triplicate and for at least three separate batches of clusters.

### Immunofluorescence analysis

For immunofluorescence analysis of whole clusters in situ, dishes were fixed in 3.7% (v/v) formalin in PBS for 10 min at RT, extracted with 0.1% triton X100 in PBS for 30 s, and then incubated in 0.5% (w/v) BSA in PBS (PBS-BSA) for 15 min to block unspecific binding. Areas surrounding clustered cells were demarked on the dish, and clusters were incubated with primary and secondary antibodies diluted in PBS-BSA essentially as described before [53]. Alternatively, clusters (approximately 10–15) were mechanically harvested from adherent monolayers and enzymatically dissociated by brief incubation in 0.1% (w/v) collagenase and 1 mg/ml dispase for 2 min at RT. After enzyme inactivation by the addition of PBS-BSA supplemented with 250 μM EDTA, cells were collected by cytospin (Thermo-Fisher Scientific, Paisley, UK), and cells were fixed and processed for immunofluorescence as described previously [25].

### Dithizone staining

The islet-like cell clusters were stained with dithizone (DTZ; Sigma-Aldrich) solution to identify the zinc-enriched insulin-expressing cells [31]. Clusters were incubated in 0.005% (w/v) DTZ in filter sterilized (0.45 μm) culture media for 15 min at 37 °C, and washed three times with warm PBS before direct microscopic examination for insulin-producing cells, which are selectively stained Crimson red.

### In vivo assay of MDSC differentiation in STZ-treated mice

Chemically induced beta-cell destruction was performed by injection of the glucose homologue streptozotocin (STZ) using the high-dose strategy (reviewed in [38]). NOD-scid-MIP-eGFP mice were fasted for 6 h prior to STZ treatment by intraperitoneal (IP) injection of a single dose of STZ at 150 mg/kg (freshly prepared in 100 mM Na-citrate pH 4.5 and used within 15 min). Mice were transplanted 1 h afterward via IP injection of 2–3 × 10^5^ MDSC stem cells in 0.2 ml saline buffer isolated from RIP-mCherry mouse muscles, or mock transplanted with IP injection of saline buffer.

### Immunofluorescence analysis on mouse pancreas

Pancreas were harvested and rinsed with PBS prior to fixation with 4% paraformaldehyde (PFA) at 4 °C for 1 h. The PFA was discarded and the pancreas washed in PBS and then kept overnight at 4 °C in 30% sucrose. For the preparation of vibratome tissue slides, the pancreas was minced into small pieces of about 0.4 mm^2^ and placed in a plastic mold. Agarose gel (4%) was cast on the tissues to provide firm holding for 150-μm sectioning (Vibratome Leica VT1000 S, Wetzlar, Germany). Alternatively, pancreas tissue fixed and equilibrated in 30% sucrose was embedded in OCT before cryosectioning (10-μm thick sections). The sections were incubated with 10% goat serum (Abcam, Cambridge, MA, USA), diluted in 0.1% Triton X-100/0.5% BSA/PBS for 1 h at room temperature, and then washed in PBS before incubation with primary antibody for 48 h at 4 °C. Subsequent incubation with secondary antibody along with Hoechst 33358 for nuclear staining was for 2 h at room temperature. Both the primary and secondary antibodies were diluted in 0.1% Triton X-100/0.5% BSA/PBS. After rinsing with PBS, sections were incubated for 5 min with 1% Sudan Black B in 70% ethanol and then washed with 70% ethanol for 5 min and distilled water for 5 min at room temperature to reduce autofluorescence.

### Blood glucose measurements in mice treated with STZ

Blood glucose levels were measured at the tail tip of 6-month-old C57Bl6 wild-type males (*n* = 8) at the same time (2 pm; corresponding to the middle of the inactive resting day time) every 3 to 4 days before and after a single IP injection of 150 mg/kg STZ in citrate buffer. When the blood glucose levels reached hyperglycemic values above 450 mg/dl, half of the mice (*n* = 4) were injected IP with 300,000 MDSC purified from the muscle of wild-type female co-sanguineous mice and glycemic levels were compared over 4 weeks in the two groups of mice.

## Additional files

Additional file 1: Figure S1. Schematic representation of the MIP-eGFP and RIP-mCherry transgenic reporter mice. Two transgenic mouse reporter models were used. The first, MIP-eGFP, contains a reporter element expressing eGFP under the control of the mouse INS1 promoter. The second, RIP-mCherry, contains monomeric mCherry under the control of the rat INS2 gene promoter. Both constructs express eGFP or mCherry in vivo exclusively in insulin-expressing beta cells in the pancreas. (JPG 150 kb)

Additional file 2: Figure S2. Cluster formation by MDSC derived from rat limb muscle. MDSC were isolated from the hind limbs of Sprague Dawley rats exactly as described for mice. After eight serial pre-plates, rat MDSC were cultured on laminin-coated dishes. Shown are phase-contrast micrographs of typical islet-like clusters formed 14 days thereafter. Scale bar = 10 μm. (JPG 483 kb)

## Abbreviations

BME: Beta-mercaptoethanol
DTZ: Dithizone
eGFP: Enhanced green fluorescent protein
ESC: embryonic stem cells
iPSC: Induced pluripotent stem cells
MDSC: Muscle-derived stem cells
MIN6: Mouse insulinoma cell line 6
MIP1: Mouse Ins-1 insulin promoter
PP: Pre-plate (day)
RIP: Rat-INS2 insulin promoter
RT: Room temperature
STZ: Streptozotocin
T1DM: Type I diabetes mellitus
