# Supplementary figures and images for: Adult muscle-derived stem cells engraft and differentiate into insulin-expressing cells in pancreatic islets of diabetic mice
Source: Stem Cell Res Ther. 2017 Apr 18;8:86. doi: 10.1186/s13287-017-0539-9 (PMC5395782; doi:10.1186/s13287-017-0539-9)

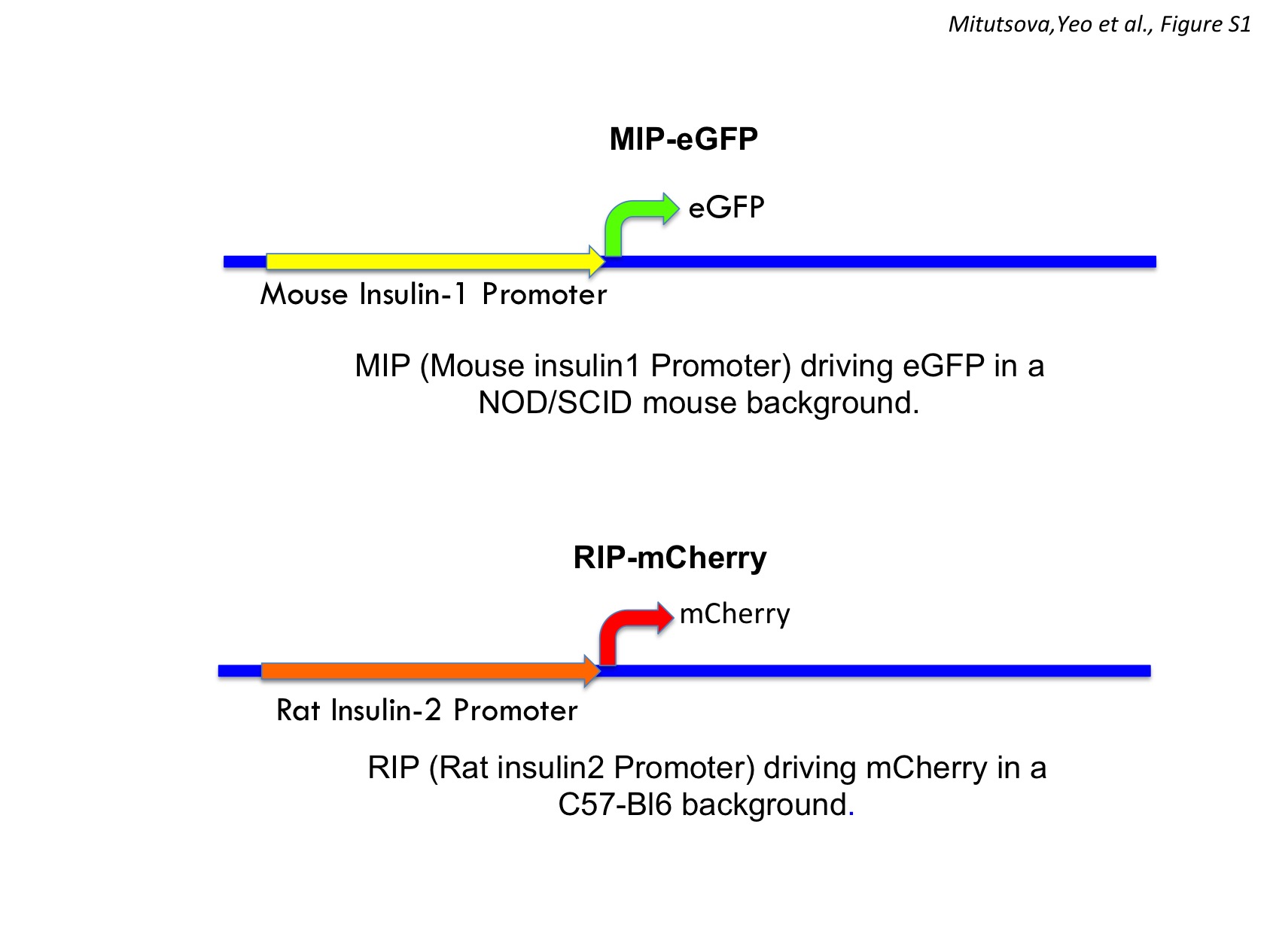

Supplement: Supplementary file 1 — Schematic representation of the MIP-eGFP and RIP-mCherry transgenic reporter mice. Two transgenic mouse reporter models were used. The first, MIP-eGFP, contains a reporter element expressing eGFP under the control of the mouse INS1 promoter. The second, RIP-mCherry, contains monomeric mCherry under the control of the rat INS2 gene promoter. Both constructs express eGFP or mCherry in vivo exclusively in insulin-expressing beta cells in the pancreas. (JPG 150 kb) [file 13287_2017_539_MOESM1_ESM.jpg]

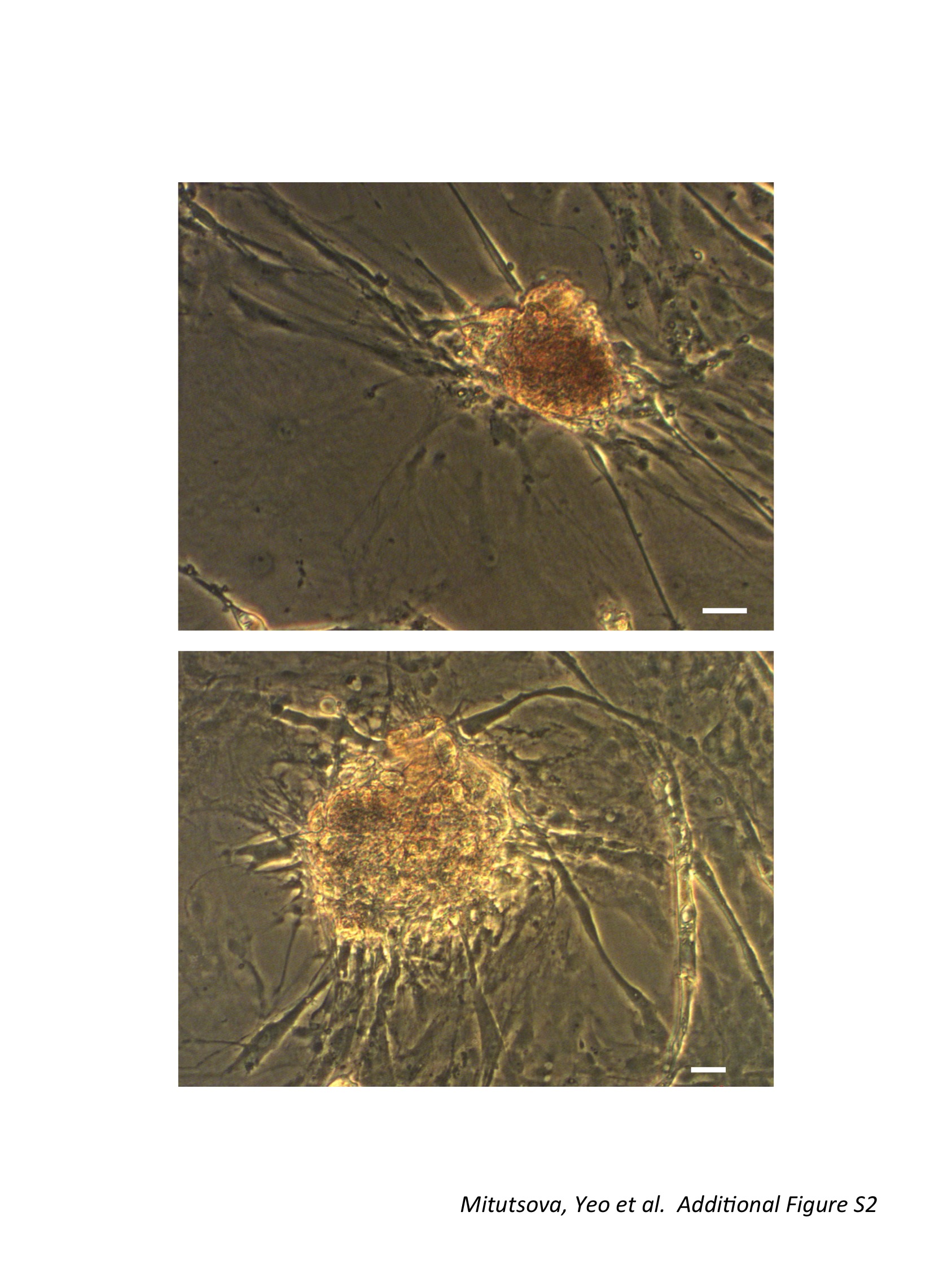

Supplement: Supplementary file 2 — Cluster formation by MDSC derived from rat limb muscle. MDSC were isolated from the hind limbs of Sprague Dawley rats exactly as described for mice. After eight serial pre-plates, rat MDSC were cultured on laminin-coated dishes. Shown are phase-contrast micrographs of typical islet-like clusters formed 14 days thereafter. Scale bar = 10 μm. (JPG 483 kb) [file 13287_2017_539_MOESM2_ESM.jpg]
